# Screening for Gram-negative bacteria: Impact of preanalytical parameters

**DOI:** 10.1038/srep30427

**Published:** 2016-07-27

**Authors:** Philipp Warnke, Friederike Pola Johanna Pohl, Guenther Kundt, Andreas Podbielski

**Affiliations:** 1 Institute of Medical Microbiology, Virology, and Hygiene, University Medicine Rostock, Rostock, Germany; 2 Institute for Biostatistics and Informatics in Medicine and Ageing Research, University Medicine Rostock, Rostock, Germany

## Abstract

Screening recommendations for multidrug-resistant Gram-negative bacteria comprise microbiological analyses from rectal swabs. However, essential specifications of the preanalytic steps of such screenings, i.e. the sampling technique, sampling devices and sampling site, are lacking. For standardized and optimum screening conditions these parameters are indispensable. Here, the optimum parameters were examined irrespective of the antibiotic resistance patterns of the target bacteria in order to establish a general basis for this type of screening. Swabs with rayon, polyurethane-cellular-foam and nylon-flocked tips were tested. Different sampling locations were evaluated, i.e. perianal, intraanal and deep intraanal. Subjects were swabbed and quantities of *E. coli*, *K. pneumoniae*, *P. aeruginosa* and *A. baumannii* were assessed. Overall prevalences of *E. coli*, *K. pneumoniae*, *P. aeruginosa*, and *A. baumannii* were 94%, 16%, 12%, and 2%, respectively. Bacterial recovery rates were independent from the sampling-timepoint during hospital stay. Polyurethane-cellular-foam or nylon-flocked swabs recovered significantly more bacteria as compared to rayon swabs. Intraanal swabbing resulted in significantly higher bacterial quantities as compared to perianal swabbing. In contrast, for the detection of *A. baumannii*, perianal swabbing seems more suitable than intraanal swabbing. Gender-related differences in bacterial recovery could be detected from perianal but not from intraanal swabs.

Infections caused by multidrug-resistant Gram-negative organisms (MDR-GNO) are associated with higher mortality rates, longer hospitalization and higher costs as compared to infections by corresponding non-resistant isolates^1^,^2^,^3^,^4^. *E. coli*, *K. pneumoniae*, *P. aeruginosa*, and *A. baumannii* are among the most frequently isolated bacteria from nosocomial infections and revealed increasing resistance rates in the last years^5^,^6^,^7^,^8^,^9^,^10^,^11^.

Recommendations on active screening are discordant. Some health authorities suggest an active screening, i.e. in Germany^12^,^13^, or the Netherlands^14^. The US Center of Disease Control (CDC) restricts active screening to the detection of carbapenem-resistant Enterobacteriaceae^15^, while the US guidelines of the Healthcare Infection Control Practices Advisory Committees (HIPAC) do not provide explicit screening recommendations^16^.

In microbiological diagnostics swab-based sample acquisition is commonly used due to its performance ease and swiftness, both from screening sites and sites of infection. Combinations of different swabs and transport systems have been evaluated for *in vitro* and *in vivo* performance data with a broad variation of results concerning the bacterial yield^17^,^18^,^19^,^20^,^21^,^22^. However, current rectal swab based screening recommendations for MDR-GNO either do not comprise such fundamental information on screening preanalysis or present contradicting information especially on the sampling locations. To achieve comparable and reliable diagnostic results, it is essential that information on the optimum sampling devices and sampling sites is given.

The present study investigates the screening performance of three widely used swab-tip materials, i.e. rayon, polyurethane cellular foam or nylon-flocked fibers. Quantitative recovery of *E. coli*, *K. pneumoniae*, *P. aeruginosa* and *A. baumannii* from three defined rectal swabbing locations, i.e. perianal, intraanal and deep intraanal, is systematically assessed. Aim of the study was to define the optimum preanalytic conditions for rectal screening in terms of recovering the highest quantities for each of the four bacterial species.

## Results

### Evaluation of swab-tip material

To investigate the optimum swab-tip material, by means of recovering the highest quantities of bacteria, subjects were swabbed with rayon, PU-foam and nylon-flocked swabs. CFUs of *E. coli*, *K. pneumoniae*, *P. aeruginosa* and *A. baumannii* were quantitatively assessed. Datasets from three different swabbing sites were included.

**Figure 1:**
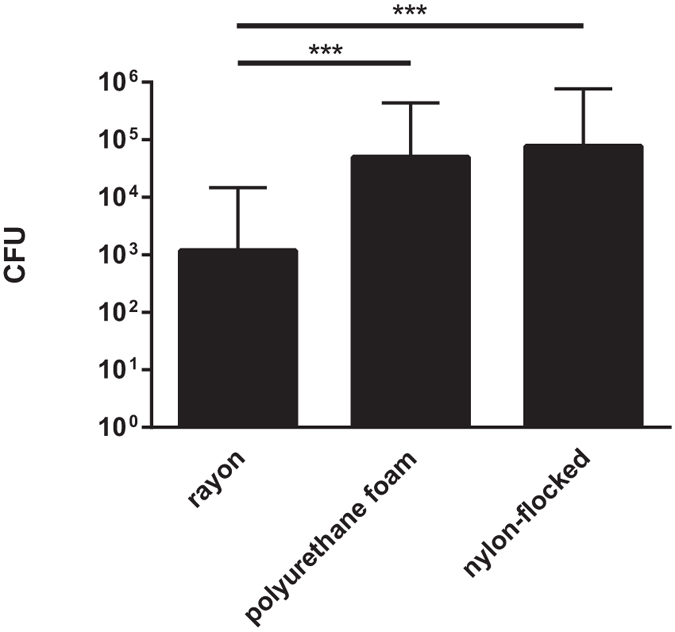
Influence of different swab-tip materials on recovered bacterial quantities. Mean quantities of *E. coli*, *K. pneumoniae*, *P. aeruginosa* and *A. baumannii* recovered from rectal swabs are displayed for different swab-tip materials. Data comprise results from three different sampling sites (perianal, 1 cm and 5 cm intraanal). CFU = colony forming units; ***p < 0.001; n = 600 datasets per column.

Highest quantities were recovered when utilizing swabs with PU-foam and nylon-flocked tips. Mean quantities of recovered bacteria varied significantly between swabs with rayon and PU-foam tips (p < 0.001), as well as between swabs with rayon and nylon-flocked tips (p < 0.001). Swabs with tips made of PU-foam and nylon-flocked fibers performed similarly with no statistically significant differences. In detail, mean recovered bacterial quantities were 1,188 CFU, 50,063 CFU and 77,015 CFU for swabs with rayon, PU-foam and nylon-flocked tips, respectively (Fig. 1).

### Evaluation of sampling sites

To analyze the impact of the sampling site on the bacterial recovery rates, subjects were swabbed at three different locations, i.e. perianally, as well as 1 cm and 5 cm intraanally. Datasets from the three different swab-types were included.

**Figure 2:**
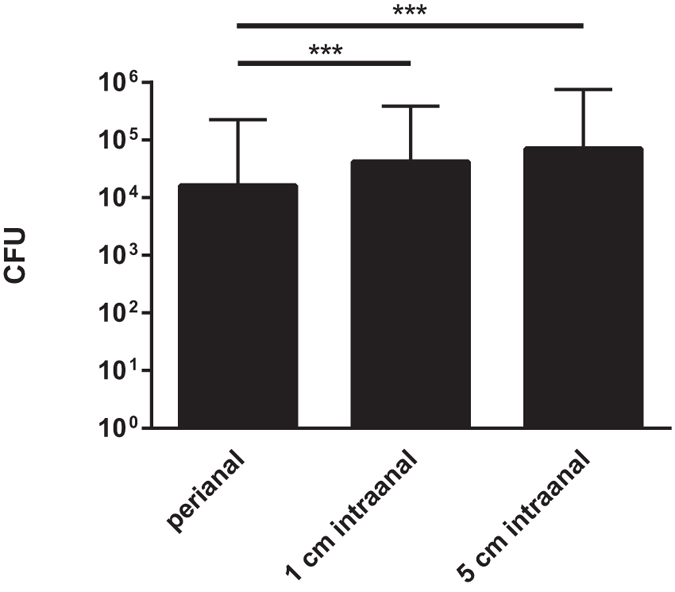
Influence of different sampling sites on recovered bacterial quantities. Mean quantities of *E. coli*, *K. pneumoniae*, *P. aeruginosa* and *A. baumannii* recovered from rectal swabs are displayed for different sampling sites. Data comprise results from three different swab-tip materials (rayon, PU-foam, nylon-flocked). CFU = colony forming units; ***p < 0.001; n = 600 datasets per column.

Highest bacterial quantities were recovered when swabbing deep intraanally. Mean quantities of recovered bacteria varied significantly between perianally and 1 cm intraanally swabbing (p < 0.001), as well as between perianally and 5 cm intraanally swabbing (p < 0.001). No statistically significant differences were observed between the two intraanal settings (p > 0.05). In detail, mean recovered bacterial quantities were 16,041 CFU, 42,039 CFU and 70,187 CFU from swabs taken perianally, 1 cm and 5 cm intraanally, respectively (Fig. 2). Analyses of the subpopulations “surgical” and “internal medicine” patients revealed similar tendencies regarding the recovered bacterial quantities (data not shown).

### Colonization sites of specific bacteria

With this study, we intended to define the optimum sampling site when in search for a specific bacterial species - a test condition that resembles the clinical situation of screening a transferred patient to verify a previously reported colonization with multidrug-resistant Gram-negative rods. Therefore, the recovered bacterial quantities for *E. coli*, *K. pneumoniae*, *P. aeruginosa* and *A. baumannii* were assessed for each sampling site. Only datasets from subjects colonized with the corresponding bacterium were included in the statistical analysis.

**Figure 3:**
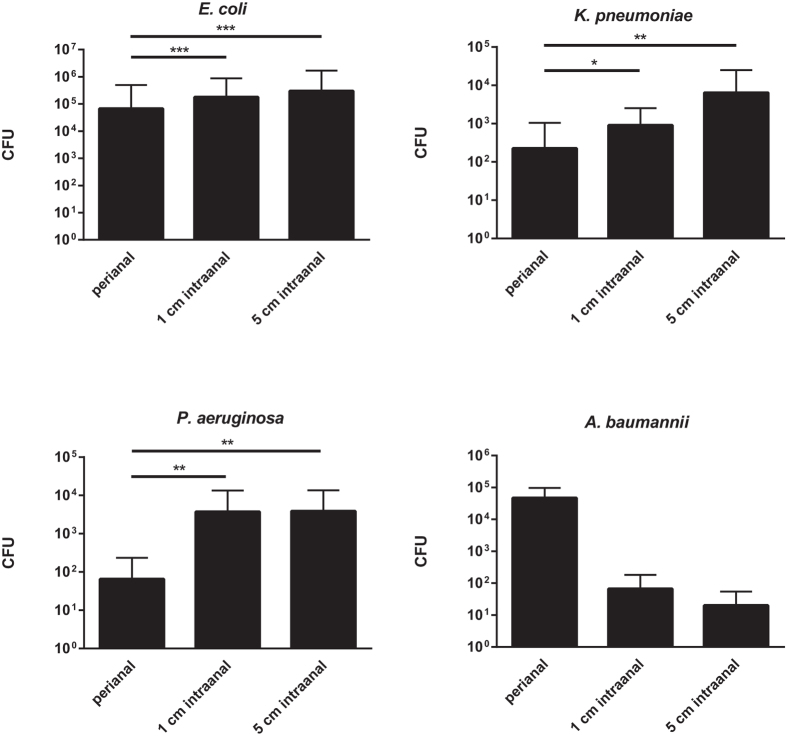
Quantities of specific bacteria recovered from different sampling sites. Mean quantities of the indicated bacterial species recovered from different sampling sites are displayed. Data comprise results from three different swab-tip materials (rayon, PU-foam, nylon-flocked). CFU = colony forming units; *p < 0.05; **p < 0.01; ***p < 0.001; n = 141; 24; 18; 3 datasets per column for *E. coli*, *K. pneumoniae*, *P. aeruginosa*, *A. baumannii*, respectively.

Compared to perianal swabbing, statistically significant higher bacterial quantities were detected intraanally for *E. coli* (p < 0.001), *K. pneumoniae* (p < 0.01) *and P. aeruginosa* (p < 0.01). For *A. baumannii* greater quantities were found perianally as compared to 1 cm or 5 cm intraanal swabbing. Due to the small number of *Acinetobacter*-colonized patients, these differences were not statistically significant (Fig. 3).

### Gender related colonization

To analyze possible gender-related differences in colonization densities, the obtained data was specifically evaluated for female and male subjects. Datasets from the three different swab-types were included.

**Figure 4:**
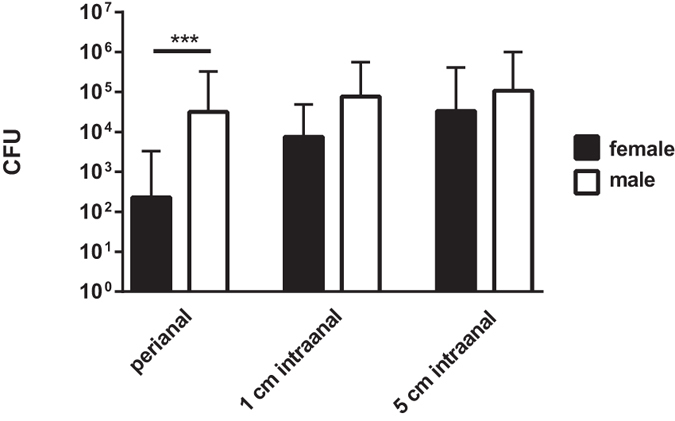
Gender-related bacterial quantities recovered from different sampling sites. Mean quantities of *E. coli*, *K. pneumoniae*, *P. aeruginosa* and *A. baumannii* recovered from rectal swabs of different sampling sites are displayed for female and male subjects. Data comprise results from three different swab-tip materials (rayon, PU-foam, nylon-flocked). CFU = colony forming units; ***p < 0.001; n = 300 datasets per column.

By intraanal swabbing no statistically significant differences concerning recovered bacterial quantities were determined between male and female subjects (p > 0.05). Perianally, statistically significant less bacterial quantities could be recovered from screening female subjects as compared to male subjects (p < 0.001) (Fig. 4).

### Influence of screening timepoints during hospital stay

To investigate the potential influence of exposure to the hospital environment on screenings results, data was evaluated for screenings performed within the first 2 days after hospital admission and compared to screenings performed thereafter. Datasets from the three different swab-types were included.

**Figure 5:**
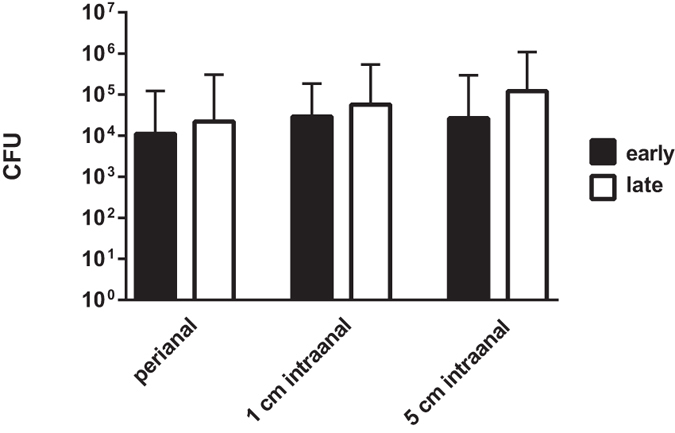
Influence of early and late performed screenings within the hospital stay. Mean quantities of *E. coli*, *K. pneumoniae*, *P. aeruginosa* and *A. baumannii* recovered from rectal swabs of different sampling sites are displayed for two sampling-timepoints within the hospital stay, i.e. early (≤2 days) and late (>2 days). CFU = colony forming units; n = 324 and 276 datasets per column for early and late analyses subsets, respectively.

Performing screenings at early or late timepoints during the hospital stay revealed no statistically significant differences concerning recovered bacterial quantities (p > 0.05) (Fig. 5).

### Prevalences of specific bacteria

To define whether perineal or rectal screening is suitable at all for the detection of *E. coli*, *K. pneumoniae*, *P. aeruginosa* and *A. baumannii*, prevalences of these species were calculated (Table 1). While *E. coli* was present in the anal region of the vast majority of our patients, *K. pneumoniae* and *P. aeruginosa* could be isolated only from smaller subsets. Of note, with respect to the extremely low number of *A. baumannii*-positive patients, for this species the anal region did not appear to be the typical place of human colonization. With respect to the timepoint of screening during the hospital, prevalences of the four bacterial species were not or only marginally affected by this parameter (Table 1).

## Discussion

Multidrug-resistant bacteria are increasingly important for daily routine handling of patients in hospitals as well as specimens in laboratories, thereby posing challenges to diagnostic, therapeutic and hygienic procedures. Thus, fast and reliable detection of these bacteria, often based on screening approaches, is crucial.

Apart from laboratory methods, for a robust and easily performable screening several points have to be addressed: (i) risk factors for carriage, (ii) appropriate transport conditions and duration, (iii) (easy accessible and well defined) screening site or body region, respectively, (iv) optimum sampling device. While risk factors for the carriage of MDR-GNO are at least partly defined^23^,^24^,^25^,^26^,^27^,^28^,^29^,^30^, publications addressing the three latter points are extremely rare or completely missing. This study addresses these latter issues and outlines the substantial impact of the swab-tip material and the swabbed region on the recovered bacterial quantities of *E. coli*, *K. pneumoniae*, *P. aeruginosa* and *A. baumannii*. A nonselective medium approach was chosen to detect all mentioned species independent from their antibiotic resistance pattern to get an insight in their favorite colonization niche.

For MSSA or MRSA the predominant colonization habitat has been identified as the anterior part of the nasal cavity^31^. This small, circumscribed and easy accessible area is extremely well suited for screening approaches. However, it was recently demonstrated that the correct sampling technique as well as the choice of an optimum sampling device could make the difference since these items significantly improved the microbial yield from MRSA screening^32^,^33^.

Obviously, different tip materials have different physical and chemical characteristics. This in turn influences the process of specimen collection and subsequent release. Therefore, concerning MDR-GNO screening, it seems prudent to first define standards, such as the optimum sampling location and sampling device, before providing general recommendations on screening.

By the present study it was demonstrated that swabs with PU-foam or nylon-flocked tips are superior in a rectal screening situation as compared to conventional rayon swabs. The better performance of these materials as compared to conventional rayon swabs is in line with findings from various clinical analyses^34^,^35^,^36^,^37^,^38^.

Furthermore, the present study revealed that deep intraanal swabbing leads to higher bacterial quantities when culturing the extracted material on solid media. This is explainable by the natural colonization habitat of the enterobacteriaceae *E. coli* and *K. pneumoniae,* i.e. the colon and rectum. Also, *P. aeruginosa* prefers moist habitats^39^,^40^,^41^. The smaller quantities of the two latter species when recovered from perianal swabs could be due to smaller numbers already encountered in the rectum. Additionally, the comparatively lower tenacity of these bacteria in the dry surface conditions present on the skin could contribute to this finding. It was demonstrated that artificially inoculated skin with *Klebsiella sp.* resulted in a loss of bacteria within 150 minutes^42^, survival in general is dependent on environmental conditions^43^,^44^. Of note, a human habitat of *A. baumannii* still highly controversial but analyses indicate a potential location rather on the skin than in the colon^25^,^45^,^46^,^47^,^48^,^49^. This goes in line with our findings on the higher quantities of *A. baumannii* recovered by swabbing from the perianal skin than from intraanal sites. Especially in an outbreak scenario or when screening transferred patients with a pre-reported MDR-GNO colonization status these colonization differences should be considered and consecutively might help to better detect a specific species by a targeted screening.

Differences between male and female subjects regarding in the bacterial quantities recovered by intraanal swabbing are not present, generally allowing for the same screening procedures for male and female patients. Differences in recovered bacterial quantities by perianal swabbing were observed, the reason remains in the range of speculation since personal hygiene habits were not examined. Regarding the investigated species-specific prevalences one could infer that rectal screening is definitely suitable for the detection of *E. coli* in subjects. It could also be appropriate when trying to detect *K. pneumoniae* and *P. aeruginosa*, even though the prevalences of the two species are rather low. With a prevalence of 2% for *A. baumannii* rectal swabbing seems to be not suitable for a reliant detection by screening in this anatomical region. Of note, within our study group, bacterial prevalences and recovered bacterial quantities were marginally affected by the length of hospital stay allowing for comparable screening results independent from sampling-timepoints.

However, differences in recovered bacterial quantities due to swab-tip materials or swabbed body regions might be bypassed when utilizing feces instead of swabs. Here, standardized amounts could be processed in the laboratory. For example, analyses for Vancomycin-resistant enterococci carriage displayed higher detection rates when analyzing stool samples compared to swab-based screenings^50^,^51^. Still, the currently available studies on a potential superiority of stool samples have to be considered with caution as they generally neither mention the employed swab types nor define the swabbed area, and finally, used semiquantitative plating methods. The differences in bacterial recovery rates between feces and different swabs have not yet been systematically investigated for MDR-GNO. Apart from the technical aspects, obtaining a stool sample from a new inpatient or from ICU patients is less plannable and therefore less well incorporable into timely diagnostic algorithms than intraanal swabs.

Additional loss of time could be due to using the wrong sampling device or swabbing at the wrong sampling site, which in turn leads to now clearly demonstrated, statistically significant lower bacterial recovery rates. As a consequence, subcultures or broth enrichments steps could be necessary, resulting in a delay to definite diagnosis of at least one day. As patients in general benefit from fast diagnostics^9^,^52^,^53^,^54^,^55^, a better recovery by choosing the right swab and swabbing at the optimum location is an important criterion.

Finally, the precise bacterial quantities detected by optimum sampling devices and locations could increase the awareness of the medical staff towards the sites most important for the transmission of the bacteria and thus, should stimulate correct hygienic behavior.

## Conclusion

This study outlines the enormous effects of swabbing devices and testing of defined body regions on the recovered bacterial quantities. Specific suggestions for optimum sampling are given. In search for MDR-GNO, screening should be performed intraanally by swabs with PU-foam or nylon-flocked tips. With this approach, gender-specific differences can be neglected. Reliable detection of *A. baumannii* by rectal swabs remains questionable. Screening recommendations must comprise precise information on screening localization, sampling devices and sampling techniques as well as transport conditions.

## Material and Methods

### Study cohort

50 subjects were included (25 male, 25 female). Inclusion criteria were age >18 years and no antibiotic treatment in the last 4 weeks. Exclusion criteria were pregnancy and inability for a written consent to this study. Patients were recruited from the following units of the Rostock University Hospital: pneumology, gastroenterology, neurology, orthopedics, urology, and surgery. Patients were examined between the first and seventh day of hospital admission (mean 2.82 d). For analyses regarding the influence of the duration of the hospital stay and the timepoint when the screening was performed, patients were separated in two groups, i.e. early screening (≤2 d of hospital stay; n = 27) and late screening (>2 d of hospital stay; n = 23).

### Swabs

The following swabs were tested:

1. Rayon tip: Sarstedt, Nuembrecht, Germany, neutral swab, via Copan, Brescia, Italy, cat. no. 80.1301.
2. Polyurethane cellular foam tip: MWE medical wire, Corsham Wiltshire England, Sigma Dry Swab Tubed, Σ-Swab, ref. MW941.
3. Nylon-flocked fiber tip: Copan, Brescia, Italy, FLOQSwabs, ref. 552C.

### Anatomical sites for screening

Swabs were obtained from three different locations: perianal, intraanal (1 cm), and deep intraanal (5 cm).

### Screening procedure

The screening was performed by the same investigator at all instances. Each screening location was swabbed by circulating while simultaneously rotating the swab for 360° and exerting gentle pressure. Each of the subjects was swabbed with each swab-type and at each anatomical site, resulting in nine swabs in total (i.e. three swab-types at three different locations). The order of the utilized swab-types was blinded/randomly chosen to avoid bias due to cyclic order. The swabbed location had to be accurately targeted and therefore one sided blinding was no option.

### Quantitative detection of microorganisms

All swabs were placed into the corresponding transport tube and were subjected to microbiological analysis within 60 minutes after rectal swabbing. Quantitative detection was performed according to CLSI quantitative swab elution method^56^. Briefly, swabs were placed in round bottom tubes (Greiner Bio-One, Frickenhausen, Germany) containing 1 ml of 0.85% isotonic saline (pH 6.8–7.2) and vortexed for 15 seconds, followed by 1:10 serial dilution steps in 0.85% isotonic saline. 100 μl of each dilution step was plated onto Columbia agar supplemented with 5% sheep blood (BD, Heidelberg, Germany), MacConkey agar (BD, Heidelberg, Germany), and CNA agar (BD, Heidelberg, Germany), respectively. Agar plates were subsequently cultured at 37 °C under a 20% O_2_/5% CO_2_ atmosphere for 48 h, followed by counting of macroscopically visible colonies (CFU).

### Identification of microorganisms

Recovered microorganisms were identified by matrix-assisted laser-desorption-ionization time-of-flight mass spectrometry (MALDI-TOF-MS) using a Shimadzu “AXIMA Assurance” MALDI-TOF mass spectrometer (Shimadzu Germany Ltd., Duisburg, Germany) as described elsewhere^57^. For MALDI-TOF analyses, isolates were prepared using alpha-cyano-4-hydroxy cinnamic acid (bioMérieux, Marcy l’Etoile, France) as matrix. Spectral fingerprints were analysed by using Vitek MS IVD V2, database MS-CE version CLI 2.0.0 (bioMérieux, Marcy l’Etoile, France).

### Statistical analysis

All data were stored and analyzed using GraphPad Prism Version 6 (GraphPad Software, San Diego, CA, USA). All p-values resulted from two-tailed, nonparametric Wilcoxon-Mann-Whitney U-test of not normal-distributed variables. Marginal-distributions between the investigated parameters were analyzed. p-values < 0.05 were considered as significant (displayed as p < 0.05 = *; p < 0.01 = **; p < 0.001 = ***). In repeated measurements analyses adjustments of alpha levels were carried out using the Bonferroni correction, i.e. the level of significance was lowered to 0.05/3 = 0.017 (for each of the three pairwise comparisons). Raw data is provided in the supplementary information (Table S1, Figure S2).

### Sample size calculation

Since expected differences and standard deviations of results from different swab-types and sampling locations were initially unknown, sample size calculation could not be performed in advance. But by measuring the primary parameter (difference of *E.coli* between swabs (averaged over locations)) three means could be calculated: 4,705 (perianal), 199,000 (1 cm), 307,000 (5 cm). Hence, the average of means was 170,000, the variance of means (V = Σ(μ_i_ − μ)^2^/3) was about 1.56*10^10^, standard deviations were 27,000 (perianal), 765,000 (1 cm), 1,360,000 (5 cm) and therefore average was 717,333. Spearman’s rho correlation between Rayon and PU foam was 0.664, between Rayon and Nylon-flocked was 0.640 and between PU foam and Nylon-flocked was 0.826, resulting in an overall average of 0.71. By using this information, sample size calculation with the nQuery, Vers. 7.0, modul MOT2-1 software and the “Univariate one-way repeated measures analysis of variance (constant correlation)” yielded n = 33 for each swab-type at a test significance level α = 0.05 and a power of 80%. The patient number was n = 42 for a power of 90%. Thus, the chosen sample size of n = 50 was well in the range of both power calculations.

According to the sample size calculations and statistical analyses, the detected statistically significant differences of the analyzed data are credible and of relevance.

### Ethics

All clinical investigation has been conducted according to the principles expressed in the Declaration of Helsinki. This study was approved by the institutional review board (IRB) of University Medicine Rostock (Ethikkommission an der Medizinischen Fakultät der Universität Rostock; approval no: A 2014–0111) in line with national and ICH-GCP guidelines. All participants provided written informed consent.

## Additional Information

**How to cite this article**: Warnke, P. *et al*. Screening for Gram-negative bacteria: Impact of preanalytical parameters. *Sci. Rep.* **6**, 30427; doi: 10.1038/srep30427 (2016).

## Supplementary Material

Supplementary Information

## Figures and Tables

**Table 1:** Combined perineal/rectal prevalences of bacteria.

	*E. coli*	*K. pneumoniae*	*P. aeruginosa*	*A. baumannii*
Prevalence (overall)	94%	16%	12%	2%
Prevalence (early; ≤ 2 days)	93%	19%	11%	0%
Prevalence (late; >2 days)	96%	13%	13%	4%

Prevalences of *E. coli*, *K. pneumoniae*, *P. aeruginosa* and *A. baumannii* recovered from different swabs and different sampling sites are displayed. Subjects were swabbed perianally, 1 cm and 5 cm intraanally with rayon, PU-foam and nylon-flocked swabs. Repeated detection from different sampling sites or swab-types within one subject was counted as one event. Prevalences are displayed for all patients irrespective of the timepoint when sampling was performed (overall) and for the subpopulations of early (≤2 days) and late (>2 days) performed screenings during the hospital stay. n = 50 subjects.
