# Supplementary material for: Screening for Gram-negative bacteria: Impact of preanalytical parameters
Source: Sci Rep. 2016 Jul 27;6:30427. doi: 10.1038/srep30427 (PMC4961960; doi:10.1038/srep30427)

Screening for Gram-negative bacteria: Impact of preanalytical parameters

Philipp Warnke, Friederike Pola Johanna Pohl, Guenther Kundt, Andreas Podbielski

Supplementary information Table S1: Raw data

Quantities of *E. coli*, *K. pneumoniae*, *P. aeruginosa* and *A. baumannii* isolates recovered from different sampling sites and swabs types. Bacterial counts are displayed as colony forming units (CFU).

	Patient	*E. coli*	*K. pneumoniae*	*P. aeruginosa*	*A. baumannii*
**Female Rayon Perianal**	1	80	0	0	0
	2	0	0	0	0
	3	90	0	0	0
	4	0	0	0	0
	5	0	0	0	0
	6	0	0	0	0
	7	70	0	20	0
	8	100	0	0	0
	9	0	0	0	0
	10	0	0	0	0
	11	0	0	0	0
	12	0	0	0	0
	13	0	0	0	0
	14	20	0	0	0
	15	0	0	0	0
	16	0	0	0	0
	17	0	0	0	0
	18	0	0	0	0
	19	0	0	0	0
	20	0	0	0	0
	21	0	0	0	0
	22	0	0	0	0
	23	0	0	0	0
	24	0	0	0	0
	25	0	0	0	0
**Male Rayon Perianal**	26	640	0	0	0
	27	0	0	0	0
	28	510	10	0	0
	29	140	0	0	0
	30	10	0	0	0
	31	0	0	0	0
	32	30	0	0	0
	33	2100	0	0	1200
	34	0	0	0	0
	35	1250	0	0	0
	36	10	0	0	0
	37	200	0	0	0
	38	240	0	0	0
	39	1000	0	0	0
	40	0	0	0	0
	41	0	0	0	0
	42	0	0	0	0
	43	0	0	0	0
	44	0	0	0	0
	45	0	0	0	0
	46	80	170	0	0
	47	0	0	0	0
	48	50	0	0	0
	49	0	0	0	0
	50	310	0	40	0

Screening for Gram-negative bacteria: Impact of preanalytical parameters

Philipp Warnke, Friederike Pola Johanna Pohl, Guenther Kundt, Andreas Podbielski

	Patient	*E. coli*	*K. pneumoniae*	*P. aeruginosa*	*A. baumannii*
**Female PU foam Perianal**	1	40	0	0	0
	2	0	0	0	0
	3	140	0	0	0
	4	0	0	0	0
	5	10	0	0	0
	6	0	0	0	0
	7	880	0	550	0
	8	2300	0	0	0
	9	0	0	0	0
	10	0	0	0	0
	11	30	10	0	0
	12	0	0	0	0
	13	0	0	0	0
	14	1300	0	0	0
	15	10	0	0	0
	16	0	0	0	0
	17	0	0	0	0
	18	0	0	0	0
	19	0	0	0	0
	20	0	0	0	0
	21	0	0	0	0
	22	53000	0	0	0
	23	0	0	0	0
	24	0	0	0	0
	25	100	0	0	0
**Male PU foam Perianal**	26	1300000	0	0	0
	27	0	0	0	0
	28	6600	0	0	0
	29	32700	0	0	0
	30	4600	0	0	0
	31	0	0	0	0
	32	330	0	0	0
	33	1280000	4000	0	40000
	34	0	0	0	0
	35	7000	0	0	0
	36	0	0	0	0
	37	350000	0	0	0
	38	1800	0	0	0
	39	40	0	0	0
	40	70	0	0	0
	41	100	0	0	0
	42	340	0	0	0
	43	0	0	0	0
	44	0	0	0	0
	45	0	0	0	0
	46	30000	800	0	0
	47	10	10	0	0
	48	1800	0	0	0
	49	0	0	0	0
	50	0	0	0	0

Screening for Gram-negative bacteria: Impact of preanalytical parameters

Philipp Warnke, Friederike Pola Johanna Pohl, Guenther Kundt, Andreas Podbielski

	Patient	*E. coli*	*K. pneumoniae*	*P. aeruginosa*	*A. baumannii*
**Female Nylon-flocked Perianal**	1	0	0	0	0
	2	0	0	0	0
	3	50	0	0	0
	4	0	0	0	0
	5	0	0	0	0
	6	0	0	0	0
	7	2300	0	500	0
	8	4200	0	0	0
	9	10	0	0	0
	10	0	0	0	0
	11	0	0	0	0
	12	0	0	0	0
	13	10	0	0	0
	14	800	0	0	0
	15	310	0	0	0
	16	0	0	0	0
	17	0	0	0	0
	18	0	0	0	0
	19	0	0	0	0
	20	0	0	0	0
	21	0	0	0	0
	22	2100	0	0	0
	23	0	0	0	0
	24	0	0	0	0
	25	10	0	0	0
**Male Nylon-flocked Perianal**	26	31000	0	0	0
	27	0	0	0	0
	28	13000	0	0	0
	29	10000	0	0	0
	30	0	0	0	0
	31	0	0	0	0
	32	30	0	0	0
	33	4530000	0	0	100000
	34	0	0	0	0
	35	3890	0	0	0
	36	0	0	0	0
	37	1490000	0	0	0
	38	600	0	0	0
	39	300000	0	0	0
	40	90	0	0	0
	41	0	0	0	0
	42	0	0	0	0
	43	0	0	0	0
	44	0	0	0	0
	45	0	0	0	0
	46	4600	380	0	0
	47	0	0	0	0
	48	70	0	0	0
	49	10	0	0	0
	50	3450	0	60	0

Screening for Gram-negative bacteria: Impact of preanalytical parameters

Philipp Warnke, Friederike Pola Johanna Pohl, Guenther Kundt, Andreas Podbielski

	Patient	*E. coli*	*K. pneumoniae*	*P. aeruginosa*	*A. baumannii*
**Female Rayon 1 cm**	1	220	0	0	0
	2	20	0	0	0
	3	80	0	0	0
	4	230	0	0	0
	5	20	0	0	0
	6	330	0	0	0
	7	0	0	170	0
	8	10	0	0	0
	9	20	0	0	0
	10	0	0	0	0
	11	400	10	0	0
	12	0	0	0	0
	13	0	0	0	0
	14	300000	0	0	0
	15	300	0	0	0
	16	0	0	0	0
	17	0	0	0	0
	18	2060	0	0	0
	19	0	0	0	0
	20	40	0	0	0
	21	0	0	0	0
	22	0	0	0	0
	23	0	0	0	0
	24	0	0	0	0
	25	110	0	0	0
**Male Rayon 1 cm**	26	109000	0	0	0
	27	60	0	0	0
	28	300	0	0	0
	29	30	0	0	0
	30	20	0	0	0
	31	0	0	0	0
	32	47000	0	0	0
	33	24600	0	0	200
	34	10	0	0	0
	35	800	0	0	0
	36	0	0	0	0
	37	0	0	0	0
	38	5600	0	0	0
	39	6100	0	0	0
	40	10	0	0	0
	41	3440	0	0	0
	42	0	0	0	0
	43	0	0	0	0
	44	0	0	0	0
	45	280	790	0	0
	46	10	10	0	0
	47	0	0	0	0
	48	50	0	0	0
	49	0	0	0	0
	50	6000	0	2400	0

Screening for Gram-negative bacteria: Impact of preanalytical parameters

Philipp Warnke, Friederike Pola Johanna Pohl, Guenther Kundt, Andreas Podbielski

	Patient	*E. coli*	*K. pneumoniae*	*P. aeruginosa*	*A. baumannii*
**Female PU foam 1 cm**	1	27000	0	0	0
	2	1460	0	0	0
	3	410	0	0	0
	4	21900	0	0	0
	5	260	0	0	0
	6	850	0	0	0
	7	6000	0	41000	0
	8	47000	0	0	0
	9	4400	20	0	0
	10	0	0	0	0
	11	12000	1000	0	0
	12	0	0	0	0
	13	110	0	0	0
	14	50000	0	0	0
	15	75000	0	3800	0
	16	0	0	0	0
	17	600	0	700	0
	18	41800	0	0	0
	19	920	0	0	0
	20	120000	0	0	0
	21	54000	0	0	0
	22	4320	0	0	0
	23	0	0	0	0
	24	0	0	0	0
	25	3400	0	0	0
**Male PU foam 1 cm**	26	353000	0	0	0
	27	130	0	0	0
	28	70000	0	0	0
	29	18500	0	0	0
	30	8300	0	0	0
	31	0	0	0	0
	32	1108000	0	0	0
	33	1500000	3000	0	0
	34	650	0	0	0
	35	3730000	0	0	0
	36	200	0	0	0
	37	700000	0	0	0
	38	550000	0	0	0
	39	100000	0	0	0
	40	317000	0	400	0
	41	230	0	0	0
	42	18800	0	0	0
	43	1100	0	0	0
	44	0	0	0	0
	45	44000	5000	0	0
	46	137000	1700	0	0
	47	10	0	0	0
	48	3500	0	0	0
	49	250	0	0	0
	50	99000	0	10000	0

Screening for Gram-negative bacteria: Impact of preanalytical parameters

Philipp Warnke, Friederike Pola Johanna Pohl, Guenther Kundt, Andreas Podbielski

	Patient	*E. coli*	*K. pneumoniae*	*P. aeruginosa*	*A. baumannii*
**Female Nylon-flocked 1 cm**	1	253000	0	0	0
	2	17940	0	0	0
	3	1680	0	0	0
	4	6800	0	0	0
	5	20	80	0	0
	6	30	0	0	0
	7	200	0	400	0
	8	150000	0	0	0
	9	44800	100	0	0
	10	10	0	0	0
	11	49000	0	0	0
	12	370000	0	0	0
	13	20	0	0	0
	14	20000	0	0	0
	15	38000	0	2100	0
	16	600	0	0	0
	17	200	0	300	0
	18	50000	0	0	0
	19	10	0	0	0
	20	32100	0	0	0
	21	400000	0	0	0
	22	3000	0	0	0
	23	0	0	0	0
	24	20	0	0	0
	25	90	0	0	0
**Male Nylon-flocked 1 cm**	26	1200000	0	0	0
	27	40	0	0	0
	28	330000	0	0	0
	29	20000	0	0	0
	30	210	0	0	0
	31	0	0	0	0
	32	1040000	0	0	0
	33	900000	1000	0	0
	34	100	0	0	0
	35	6830000	0	0	0
	36	180	0	0	0
	37	1630000	0	0	0
	38	60000	0	0	0
	39	620000	0	0	0
	40	690000	0	3000	0
	41	100	0	0	0
	42	13500	0	0	0
	43	50	0	0	0
	44	0	0	0	0
	45	12000	5000	0	0
	46	550000	4000	0	0
	47	140	0	0	0
	48	3000	0	0	0
	49	1000	0	10	0
	50	58200	0	2800	0

Screening for Gram-negative bacteria: Impact of preanalytical parameters

Philipp Warnke, Friederike Pola Johanna Pohl, Guenther Kundt, Andreas Podbielski

	Patient	*E. coli*	*K. pneumoniae*	*P. aeruginosa*	*A. baumannii*
**Female Rayon 5 cm**	1	1640	0	0	0
	2	20	0	0	0
	3	30	0	0	0
	4	30	0	0	0
	5	0	0	0	0
	6	60	0	0	0
	7	2500	0	200	0
	8	90	0	0	0
	9	5700	20	0	0
	10	0	0	0	0
	11	5300	60	0	0
	12	380	0	0	0
	13	10	0	0	0
	14	10	0	0	0
	15	10	0	0	0
	16	0	0	0	0
	17	0	0	0	0
	18	810	0	0	0
	19	0	0	0	0
	20	44000	0	0	0
	21	80	0	0	0
	22	0	0	0	0
	23	0	0	0	0
	24	0	0	0	0
	25	900	0	0	0
**Male Rayon 5 cm**	26	40	0	0	0
	27	0	0	0	0
	28	0	0	0	0
	29	30	0	0	0
	30	0	0	0	0
	31	0	0	0	0
	32	52800	0	0	0
	33	5200	60	0	60
	34	12000	0	0	0
	35	17300	0	0	0
	36	0	0	0	0
	37	0	0	0	0
	38	0	0	0	0
	39	36500	0	0	0
	40	470	0	0	0
	41	480	0	0	0
	42	0	0	0	0
	43	0	0	0	0
	44	0	0	0	0
	45	1900	1800	0	0
	46	0	30	0	0
	47	0	0	0	0
	48	40	0	0	0
	49	0	0	0	0
	50	3280	0	90	0

Screening for Gram-negative bacteria: Impact of preanalytical parameters

Philipp Warnke, Friederike Pola Johanna Pohl, Guenther Kundt, Andreas Podbielski

	Patient	*E. coli*	*K. pneumoniae*	*P. aeruginosa*	*A. baumannii*
Female PU foam 5 cm	1	41000	0	0	0
	2	1690	0	0	0
	3	300	0	0	0
	4	1960	0	0	0
	5	30	140	0	0
	6	1000	0	0	0
	7	10000	0	40000	0
	8	62000	0	0	0
	9	31000	50	0	0
	10	560	0	0	0
	11	47000	2000	0	0
	12	790	0	0	0
	13	50	0	0	0
	14	810000	0	0	0
	15	33600	0	2700	0
	16	20	0	0	0
	17	100	0	300	0
	18	280000	0	0	0
	19	110	0	0	0
	20	48000	0	0	0
	21	140000	0	0	0
	22	10300	0	0	0
	23	0	0	0	0
	24	0	0	0	0
	25	12400	0	0	0
Male PU foam 5 cm	26	285000	0	0	0
	27	1170	0	0	0
	28	31000	0	0	0
	29	10000	0	0	0
	30	15800	0	0	0
	31	0	0	0	0
	32	4600000	0	0	0
	33	3250000	2000	0	0
	34	650	0	0	0
	35	6160000	0	0	0
	36	30	0	0	0
	37	2310	0	0	0
	38	550000	0	0	0
	39	160000	0	0	0
	40	330000	0	800	0
	41	810	0	0	0
	42	24400	0	0	0
	43	0	0	0	0
	44	0	0	0	0
	45	100000	50000	0	0
	46	250000	2100	0	0
	47	0	0	0	0
	48	330	0	0	0
	49	10	0	0	0
	50	211000	0	7000	0

Screening for Gram-negative bacteria: Impact of preanalytical parameters

Philipp Warnke, Friederike Pola Johanna Pohl, Guenther Kundt, Andreas Podbielski

	Patient	*E. coli*	*K. pneumoniae*	*P. aeruginosa*	*A. baumannii*
**Female Nylon-flocked 5 cm**	1	26000	0	0	0
	2	1800	0	0	0
	3	22000	0	0	0
	4	24400	0	0	0
	5	0	0	0	0
	6	50	0	0	0
	7	15000	0	15000	0
	8	150000	0	0	0
	9	6300000	5000	0	0
	10	390	0	0	0
	11	17000	3000	0	0
	12	7400	0	0	0
	13	4800	0	0	0
	14	119000	0	0	0
	15	4500	0	0	0
	16	0	0	0	0
	17	600	0	270	0
	18	1430000	0	0	0
	19	0	0	0	0
	20	76800	0	0	0
	21	190000	0	0	0
	22	24800	0	0	0
	23	0	0	0	0
	24	10	0	0	0
	25	310	0	0	0
**Male Nylon-flocked 5 cm**	26	13000	0	0	0
	27	0	0	0	0
	28	830000	0	0	0
	29	5100	0	0	0
	30	19800	0	0	0
	31	0	0	0	0
	32	62000	0	0	0
	33	13000000	80000	0	0
	34	160	0	0	0
	35	20900	0	0	0
	36	60	0	0	0
	37	10200	0	0	0
	38	45000	0	0	0
	39	870000	0	0	0
	40	190000	0	500	0
	41	80000	0	0	0
	42	19900	0	0	0
	43	120	0	0	0
	44	0	0	0	0
	45	14000	7000	0	0
	46	4400	220	0	0
	47	190	10	0	0
	48	520000	0	0	0
	49	13000	0	450	0
	50	50000	0	2400	0

Screening for Gram-negative bacteria: Impact of preanalytical parameters

Philipp Warnke, Friederike Pola Johanna Pohl, Guenther Kundt, Andreas Podbielski

Supplementary information Figure S2: Matches / discrepancies between swab-types

Matches / discrepancies, i.e. positive or negative results for the detection of *E. coli*, are displayed for each swab type at each sampling site.

Perianal

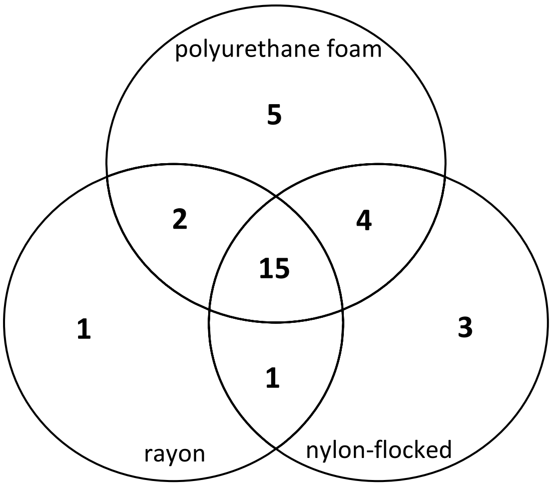

1 cm intraanal

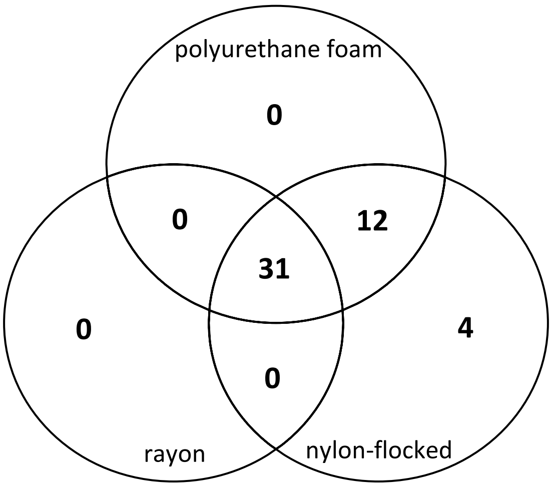

5 cm intraanal

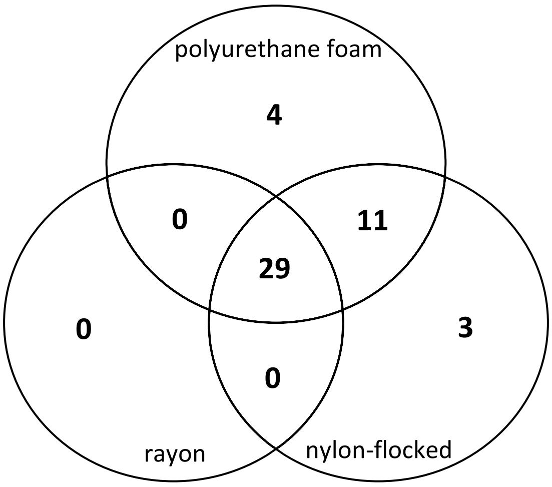

Supplement: Supplementary Information [file srep30427-s1.pdf]
